# Minding mentalizing - convergent validity of the Mentalization Breakdown Interview

**DOI:** 10.3389/fpsyt.2024.1380532

**Published:** 2024-06-21

**Authors:** Dag Anders Ulvestad, Merete Selsbakk Johansen, Elfrida Hartveit Kvarstein, Geir Pedersen, Theresa Wilberg

**Affiliations:** ^1^ Outpatient Clinic for Specialized Treatment of Personality Disorders, Section for Personality Psychiatry and Specialized Treatments, Department for National and Regional Functions, Division of Mental Health and Addiction, Oslo University Hospital, Oslo, Norway; ^2^ Institute of Clinical Medicine, University of Oslo, Oslo, Norway; ^3^ South-Eastern Norway Regional Health Authority, Oslo, Norway; ^4^ Section for Personality Psychiatry and Specialized Treatments, Department for National and Regional Functions, Division of Mental Health and Addiction, Oslo University Hospital, Oslo, Norway; ^5^ Network for Personality Disorders, Section for Personality Psychiatry and Specialized Treatments, Department for National and Regional Functions, Division of Mental Health and Addiction, Oslo University Hospital, Oslo, Norway; ^6^ Institute of Basic Medical Sciences, University of Oslo, Oslo, Norway; ^7^ Section for Treatment Research, Department for Research and Innovation, Division of Mental Health and Addiction, Oslo University Hospital, Oslo, Norway

**Keywords:** borderline personality disorder, mentalizing, mentalization breakdown, reflective functioning, assessment method, validation

## Abstract

**Objectives:**

Mentalizing difficulties are central to borderline personality disorder (BPD), have severe consequences, and are an explicit focus in mentalization-based treatment. The significance of mentalizing capacity as a predictor or mediator of change is however still uncertain due to a scarcity of research. The Mentalization Breakdown Interview (MBI) was developed as a time saving tool for studying psychotherapy processes and outcome in borderline pathology. This study aimed to investigate the convergent validity of reflective functioning (RF) ratings based on the MBI (MBI-RF) by a comparison with the gold standard, i.e., RF assessments based on the Adult Attachment Interview (AAI-RF). A secondary aim was to investigate how MBI-RF relates to core symptoms of BPD, levels of functional impairment and symptom distress compared with AAI-RF.

**Method:**

Forty-five patients with BPD or significant BPD traits were included. MBI-RF and AAI-RF were rated using the Reflective Functioning Scale. Levels of MBI-RF and AAI-RF and the correlation between the measures were calculated, as well as their associations with the Difficulties in Emotion Regulation Scale, Levels of Personality Functioning-Brief Form 2.0, Work and Social Adjustment Scale, Patient Health Questionnaire, Depression, Generalized Anxiety Disorder-7, self-harm, suicide attempts, and PD diagnostics.

**Results:**

The correlation between MBI-RF and AAI-RF was 0.79 (*p*<0.01), indicating high convergent validity. There were few significant associations between MBI-RF and AAI-RF and clinical measures.

**Conclusions:**

The study provides support for the convergent validity of the MBI as a BPD-focused RF assessment method. The MBI has the potential as a time saving, reliable and valid method to be applied in treatment research on patients with borderline pathology. The results indicate that measures of MBI-RF and AAI-RF are different from clinical symptoms.

**Clinical trial registration:**

ClinicalTrials.gov ID NCT04157907.

## Introduction

Mentalizing is defined as the imaginative mental activity that enables us to perceive and interpret human behavior in terms of intentional mental states like beliefs, thoughts, and feelings (1).

In recent decades, mentalizing has found its place as a transdiagnostic concept both in therapeutic practice and in psychotherapy research, and with a particularly prominent role for borderline personality disorder (BPD) (2). However, despite an assumption that improvement in mentalizing capacity is associated with positive treatment outcome in BPD, the evidence base is still limited and to some extent inconclusive (3–5).

Originally, the operationalization of mentalization capacity has been through the assessment of reflective functioning (RF) (6). The gold standard for assessment of RF is the application of the Reflective Functioning Scale (RFS; 7) on the Adult Attachment Interview (AAI) (8). The RFS applied on the AAI has been extensively used in psychotherapy research, and in a recent scoping review this measure of mentalization was the only observer-based instrument proven to be sensitive to change (2). However, the administration, transcribing and coding of this interview makes it a time consuming and costly method that hampers its use in research on treatment processes (9, 10).

Consequently, in the majority of treatment studies using AAI-based RF (AAI-RF), only pre- and post-assessments are applied (2). A range of other RF assessment methods have therefore been developed, primarily self-report measures, intended to reduce administration time and increase applicability (11–16). However, the validity of self-report measures has been questioned (17), and several potential limitations of self-report measures has been emphasized, including lack of meta-perspective, social desirability bias, and possibilities of misinterpretation of the questions (14, 18). In patients with personality disorders (PDs) characterized by egosyntonic traits and significant impairments in self-reflexivity, scores based on self-report can be even more challenging to the validity of inferences (19).

Among the interview-based instruments a different approach to assess mentalizing ability is symptom-specific RF interviews. Currently, such interviews exist for obsessive-compulsive disorder, panic disorder, post-traumatic stress disorder and depression (20, 21). Despite the focus on current specific condition or symptoms this approach can maintain the complexity of the original RF concept, as long as the questions probe for RF in the same manner as demand questions (i.e., questions which demand a demonstration of mentalizing capacity) in the AAI (22). A core feature of BPD is significantly impaired mentalizing capacity, sometimes evolving into complete breakdowns, potentially leading to severe relational problems, self-destructive behavior, violence, or substance misuse (23, 24). Until recently, there were no RF assessment methods that specifically focus on BPD patients’ capacity to reflect on mentalizing breakdowns in current close relationships. The Mentalization Breakdown Interview (MBI) was thus developed as a BPD-focused method to supplement existing RF assessment methods (25). While also requiring less resources, it represents a potential tool in further research on the mechanisms of change in the treatment of borderline personality pathology. However, the convergent validity of this new RF assessment method remains to be established.

Moreover, regarding how interview-based RF relates to core symptoms and functioning in BPD, the research is limited and inconsistent. A study among female BPD patients found no relation between AAI-RF and number of comorbid Axis I and Axis II diagnoses (26). In a mixed clinical sample of avoidant PD and BPD Antonsen et al. (24) found that low AAI-RF was associated with more self-reported symptom distress, psychosocial impairment and personality difficulties in the self- and relational domains. There were however no differences between patients with respectively low or medium RF scores regarding number of PD criteria or Axis I diagnoses. The study did not investigate the BPD patients specifically. In a mixed PD (82% with BPD) and non-psychiatric sample AAI-RF was negatively correlated with self-reported distress (27), while at two-year follow up improvement in AAI-RF were significantly associated with improved social adjustment and global functioning but not with improvement in psychiatric symptom distress (5). Moreover, in a cross-sectional study of females with or without BPD the variance in global function explained by RF was modest compared to the influence of BPD severity, childhood sexual trauma and socio-economic factors (28). Clearly, more studies are needed to explore how interview-based RF, like AAI-RF and MBI-RF, relates to borderline personality pathology.

## Aims

The primary aim of the present study was to investigate the convergent validity of RF ratings based on the MBI by comparing MBI-RF with AAI-RF. A secondary aim was to investigate how MBI-RF relates to core symptoms of BPD, levels of functional impairment and symptom distress compared with AAI-RF. Our research questions were: 1) how strong is the linear relationship between MBI-RF and AAI-RF? And 2) what are the associations between the two RF-measures and psychosocial functioning, level of personality functioning, emotion regulation, self-harm, suicidal behavior, and PD diagnostics?

## Materials and methods

### Study setting

The study comprises baseline data from 45 patients included in the project *“Reflective functioning and psychotherapy processes in Mentalization Based Therapy (MBT)”*. The study was performed at Oslo University Hospital in an outpatient clinic specialized in treating patients with BPD. The treatment offered was MBT (29), and the study involved regular patients and clinicians who applied the MBI as part of the assessment practice (25).

### Procedures

The clinical staff at the unit performed the MBIs as part of the initial evaluation for treatment, whereas two specially trained researchers (TW & external researcher) conducted the AAIs. At similar time point, the patients filled in self-report questionnaires and the clinicians performed diagnostic evaluation according to the DSM-5 (30) using respectively the Structured Clinical Interview for DSM-5 Personality Disorders for PDs (SCID-5-PD) (31) and the Mini International Neuropsychiatric Interview (MINI) (32) for symptom disorders. A specialist in psychiatry or clinical psychology at the unit confirmed diagnostic classification. Patients with schizophrenia, schizoaffective disorder, bipolar disorder type I, alcohol or substance dependency, autism spectrum disorder or cognitive impairment were not included in the treatment program. In addition the clinicians rated the patients on the revised version of the Global Assessment of Functioning Scale (GAF); the Global Functioning Scale (GFS) (33, 34). Conventional interpretations of severity indicated by GFS scores are similar to the original GAF: Mild (61–70); Moderate (51–60); and Severe (41–50) (33).

### Participants

Only patients with four or more fulfilled BPD-criteria were included in the study. Sociodemographic, clinical and diagnostic status is reported in **Tables 1**, **2**. Average age of the participants was 23 (SD=3), ranging from 19–30 years. The sample comprised five men (11%) and 40 women (89%). Global functioning (GFS) indicated moderate to severe impairment (M=51.0, SD=4.0). The level of education was relatively low (M=3.9, SD=2.5) (**Table 1**). Participation in work or studies was also low, with a sample average of 3.0 months (SD=2.7) participation at least half-time in work or study activity last six months.

**Table 1:** Clinical and demographic status at initial assessment, n=45.

	Percent	Mean (SD)
Demographics		
Age		23.2 (3.4)
Female	89	
Cohabiting or married	20	
Years education after mandatory school (age 6–16)		3.9 (2.5)
Months > 50% work/study last six months		3.0 (2.7)
Former treatment experience		
Previous treatment in mental health services	84	
More than two treatment series	49	
First treatment < 18 years of age	71	
Previous hospital admissions	47	
Functioning		
Global functioning (GFS)		51.0 (4.0)
Work and social impairment (WSAS)		24.0 (7.1)
Level of personality functioning, Total (LPFS-BF Total)		21.8 (5.3)
Level of personality functioning, Self (LPFS-BF Self)		13.0 (2.9)
Level of personality functioning, Other (LPFS-BF Other)		8.8 (3.2)
Symptom distress		
Depression (PHQ-9)		20.6 (3.7)
Anxiety (GAD-7)		13.6 (5.1)
Emotional dysregulation (DERS)		
Clarity		3.5 (0.9)
Awareness		3.0 (0.8)
Non-acceptance		4.0 (1.0)
Goals		4.2 (0.6)
Impulses		3.6 (0.9)
Strategies		3.9 (0.7)
Self-harming and suicide attempts		
Self-harm, lifetime	89	
Self-harm < 13 years of age	40	
Self-harm, last 6 months	78	
Self-harm, daily or weekly last 6 months	34	
Suicide attempt, lifetime	68	
Suicide attempt < 13 years of age	7	
Suicide attempt, last 6 months	19	

GFS, Global Functioning Scale; WSAS, Work and Social Adjustment Scale; PHQ-9, Patient Health Questionnaire, Depression; GAD-7, Generalized Anxiety Disorder-7; LPFS-BF, The Levels of Personality Functioning- Brief Form; DERS, Difficulties in Emotion Regulation Scale.

**Table 2:** Diagnostic status for patients after initial assessment.

	Percent	Mean (SD)
Personality disorders		
Schizoid & Schizotypal	0	
Paranoid	2	
Antisocial	0	
Narcissistic & Histrionic	0	
Borderline	93	
Avoidant	13	
Dependent	2	
Obsessive Compulsive	4	
PD NOS	7	
Severity of personality difficulties		
Total number of SCID-5-PD criteria		13.6 (5.3)
Number of BPD criteria		6.3 (1.4)
Number of PD diagnoses		1.2 (0.6)
Symptom disorders		
Major depression	33	
PTSD	20	
Agoraphobia with panic disorder	13	
Eating disorder NOS	11	
GAD	7	

PD NOS, Personality disorder not otherwise specified; Eating disorder NOS, Eating disorder not otherwise specified.

Most patients (93%) had five or more BPD criteria (M=6.3, SD=1.4), thus qualifying for a diagnosis of BPD, and the most common co-occurring PD was avoidant PD (13%) (**Table 2**). The vast majority had one PD diagnosis (84%), while 9% had two and 7% had three. The total number of PD criteria were in the range 7–29 (M=13.6, SD=5.3).

Regarding symptom disorders, average number of diagnoses were 2.3 (SD=1.2). Current major depression was most frequent (33%), followed by PTSD (20%), agoraphobia with panic disorder (13%), eating disorder not otherwise specified (11%), and generalized anxiety disorder (7%). Most patients (89%) had a history of self-harm, and 40% had self-harmed before the age of 13. Sixty-eight percent had previously made one or more suicide attempts, wherein 19% during the last six months. The majority (84%) also had previous treatment for psychiatric symptoms, and 47% had previously been admitted to a psychiatric ward.

In sum, the participants had significant impairments in work and social functioning, a severe burden of symptom distress, self-harming behavior and suicide attempts. The contact with the healthcare system was established early on and it was a fairly extensive use of healthcare services.

### MBI training

The majority of the clinicians performing the MBIs had extensive training and education in MBT, and all interviewers were familiar with the theory of mentalizing and MBT and received weekly, video-based MBT supervision. Before and during the study period the clinicians also attended a series of group-based training sessions concerning the administration of the MBI to ensure and maintain interview competence (25).

### Raters & rating procedures

Three of the authors (MSJ, TW & DAU) are certified as reliable RF coders. They rated video recorded MBIs for MBI-RF. The raters were blind to all identifying characteristics of the participants. -Two of the authors (TW & DAU) rated the AAIs for AAI RF based on verbatim transcripts of the interviews. TW rated 13 of the AAIs conducted by herself, wherein seven were part of the reliability test.

### Assessments

*The Mentalization Breakdown Interview (MBI)* (25) is a semi-structured 30-minute interview that can be conducted by a clinician with knowledge of the theory of mentalizing and MBT. The interview is an extension of a clinical interview guide originally developed by prof. Sigmund Karterud and published in a MBT manual (35, 36, pp. 78–82). The further development of the interview for research purposes was inspired by the studies of symptom-specific RF interviews (20). When conducting the MBI an initial explanation of the purpose of the interview is given to the interviewee. Then the interviewee is asked if he/she has experienced mentalizing breakdowns and whether these are typical to their life. Thereafter a recent and preferably severe episode (last six months) of mentalizing breakdown in a close relationship is selected for exploration. The course of events, interpersonal context, triggers and consequences of the breakdown episode are clarified before an in-depth exploration is undertaken. Based on video recording of the interview a global RF-score is rated using the RFS. RFS is an eleven-point scale ranging from – 1 (anti-reflective) to + 9 (exceptional reflective) (7). The questions in the MBI are divided into those that permit and those that demand a demonstration of mentalizing capacity. There are respectively four demand questions and two permit questions which are scored separately before a global score is given. See Ulvestad et al. (25)for a more detailed description of the questions in the interview. Responses to the demand questions constitute the basis for the scoring, while the responses to the permit questions provide incremental information when assigning a global score (22). The MBIs (n=44) were respectively scored in pairs (n=24) and by all three raters (n=20), and for each interview a consensus score was agreed upon. The inter-rater reliability (IRR) was estimated by the Intra-class Correlation Coefficient for Two-way random effects, average measure, consistency (ICC, 2.k). In a previous study the ICC of MBI ratings for this group of raters was found to be good (ICC, 2.k; 0.77, 95% C.I.: 0.59 -0.88) (25). In the present study the ICC for the group of three raters was 0.81 (95% C.I.: 0.61–0.92), while the coefficients for the pairwise groups were respectively 0.69 (95% C.I.: 0.35–0.85), 0.72 (95% C.I.: 0.44–0.86) and 0.84 (95% C.I.: 0.65–0.93) indicating moderate to good reliability.

*The Adult Attachment Interview (AAI)* is a semi-structured interview consisting of 23 specified questions in addition to situation-specific probes addressing the individual’s experiences of childhood relationships with attachment figures, as well as their influences on the individual as an adult. Eight of the specified questions are demand questions, while fifteen are permit questions. The interview lasts about an hour. The RFS is applied to a verbatim transcript of the interview resulting in a global RF-score (7). AAI-RF has been shown to be a valid and reliable measure of mentalizing capacity in non-clinical as well as clinical samples, also including BPD-samples (7, 22, 37). A total of 45 AAIs were scored, and IRR was established on a subset of 7 randomly chosen interviews. IRR was estimated by the Intra-class Correlation Coefficient for Two-way random effects, average measure, consistency (ICC, 2.k). The ICC for the AAIs were 0.85 (95% C.I.: 0.27–0.97), indicating good reliability (38). For the interviews that were part of the reliability analysis a consensus score was agreed upon, to be used in the further data analyses.

*The Difficulties in Emotion Regulation Scale (DERS)* (39) is a self-report questionnaire assessing emotional awareness and regulation. It comprises 36-items rated on a 1–5 scale. Higher scores suggest greater problems with emotion regulation. Items are organized in 6 facets: Lack of Emotional Clarity (Clarity); Lack of Emotional Awareness (Awareness); Non-acceptance of Emotional Responses (Non-acceptance); Difficulties Engaging in Goal-Directed Behavior (Goals); Impulse Control Difficulties (Impulse); and Limited Access to Emotion Regulation Strategies (Strategies). DERS has been shown to be a valid and reliable measure of emotional dysregulation (39, 40). Moreover, in a sample of 2302 psychiatric outpatients in treatment for PD or personality related problems, scale reliability of the DERS subscales ranged from .78 (Awareness) to .90 (Impulse) (41).

*The Levels of Personality Functioning-Brief Form, second version (LPFS-BF 2.0)* is a patient self-report based on the DSM–5 AMPD (Alternative Model of Personality Disorders, Section III) (42) with 12 items clustered in two domains; self-functioning (Self) and interpersonal functioning (Other). It is rated on a 0–3 scale, sum-score ranges 0–36, where higher scores indicate more impaired personality functioning. However, the measurement level vary among studies of LPFS-BF, in that some use a 0–3 response format, whereas other use a 1–4 point format. In a German normative study, using a 1–4 point format, estimated average sum score on LPFS-BF was 15 (SD=9) (43). Correspondingly, in a Danish population study, also using a 1–4 format, a sum score of 26 indicates mild or subclinical dysfunction, a score of 31 indicates moderate level of dysfunction, 36 a severe clinical dysfunction, and that sum scores at or above 41 indicate extreme dysfunction (44). For studies using a 0–3 response scale the corresponding thresholds proposed by Weekers et al. (44) is obtained by subtracting the scores by 12, which gives corresponding thresholds of 14, 19, 24, and 29, respectively. In a recent study with comparable setting as the present study, scale reliability for a sample of patients with PD or personality related problems was found to be satisfactory (45).

*Generalized Anxiety Disorder-7 (GAD-7)* is a patient self-report questionnaire with seven items rated on a four-point (0–3) response format (46). Sum scores ≥10 indicate a possible generalized anxiety disorder (47). Scale reliability has been found satisfactory in a sample of patients with PD or personality related problems (48).

*Patient Health Questionnaire, Depression (PHQ-9) is* a self-report questionnaire with nine items rated on a four-point (0–3) response format (49). Sum scores ≥10 indicate clinically relevant depressive symptoms (50). Scale reliability has been found satisfactory in a sample of patients with PD or personality related problems (48).

*Work and Social Adjustment Scale* (*WSAS*) (51), is a self-report questionnaire of five items rated on a nine-point response format from 0 (no impairment) to 8 (extreme impairment). The items cover the following social aspects: Ability to work or study, home management, social leisure activities, private leisure activities, and close relationships. Mild-to-no impairment is indicated by sum scores <15, moderate–severe impairment by sum scores 15–30, and extreme impairment by sum scores >30. The WSAS is considered reliable and clinically relevant in PD samples (48, 52).

*Self-harm and suicide attempts*. History of self-harm and suicide attempts were assessed during the initial assessment. Self-harm was measured by self-report questions (Yes/No), with reference to occurrence lifetime and past six months: “Have you ever intentionally harmed yourself (cut/scratched/burned/scalded yourself, hit your head against the wall, or similar)?” and “In the past six months, have you intentionally harmed yourself?” Suicide attempts were measured accordingly, addressing both lifetime and past six months: “Have you ever made a suicide attempt?” and “In the past six months, have you made a suicide attempt?” Further, age first time and number of incidents of self-harm and suicide attempt were addressed, as well as frequency of self-harm last six months.

### Statistics

Paired and independent samples t-tests were used when analyzing level- and group-differences, while Pearson product-moment correlation was used for linear associations. We applied an alpha-level of 0.05. In planning the study, power calculation estimated that a sample size of 45 would enable detection of significant correlation coefficients from 0.40, with power 0.8 given an alpha=0.05 (http://psychstat.org). All analyses were performed using SPSS Statistics for Windows, Version 29 (53).

## Results

**Figure 1:**
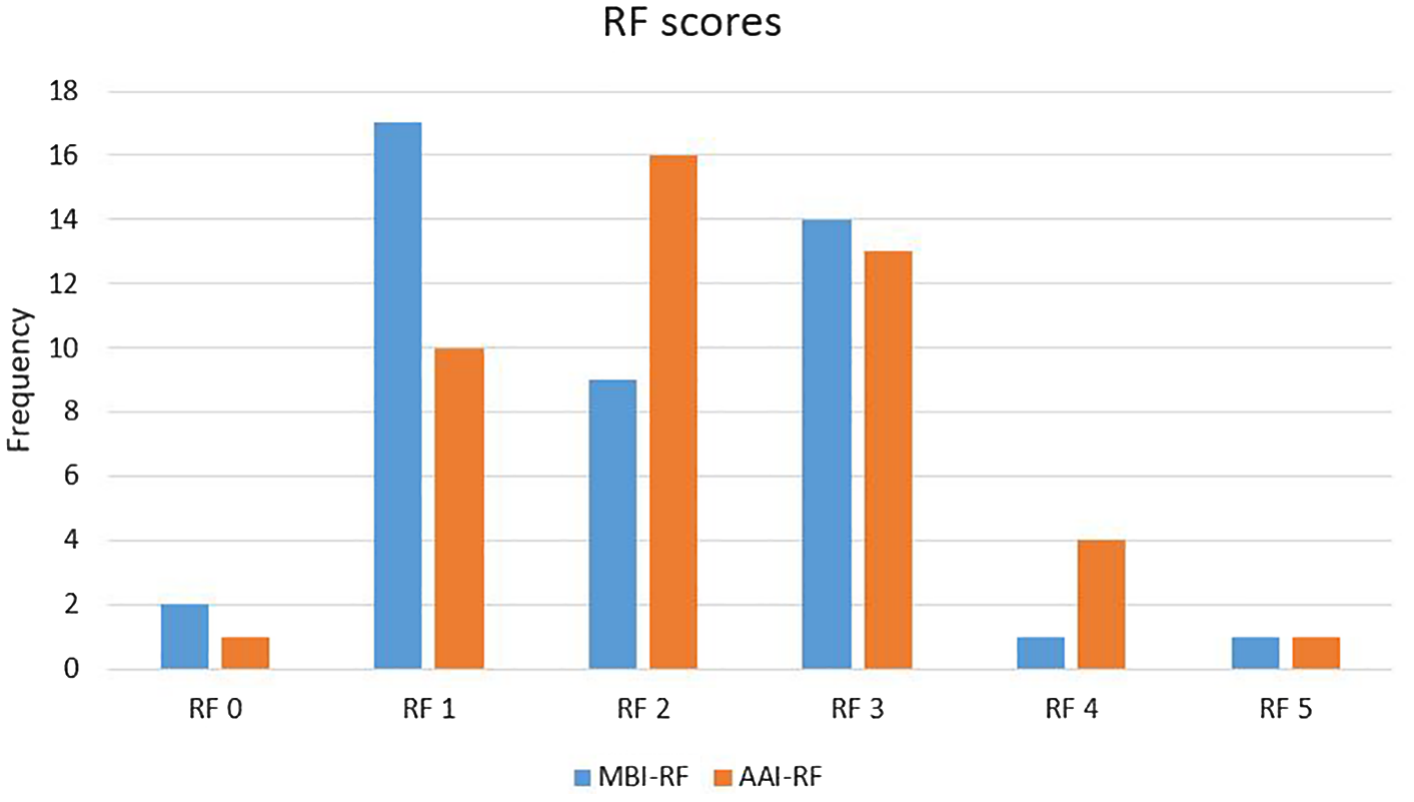
Distribution of RF scores on the MBI and the AAI.

The mean level of RF was 1.95 (SD=1.10) for MBI and 2.27 (SD=1.05) for AAI. The difference was statistically significant (t=2.79, *p*=0.008). The RF scores were in the range 0–5 for both MBI-RF and AAI-RF, with the majority of scores in the range 1–3 (see **Figure 1**).

The RF scores on the MBIs and the AAIs were checked for potential bias due to sex and age. There was a positive correlation between MBI-RF and age (*r*=0.31, *p*=0.044). Otherwise, no indication of bias regarding sex and age was found in the sample.

The correlation (Pearson’s *r*) between MBI-RF and AAI-RF was 0.79 (*p*<0.01), indicating high convergent validity between the measures.

As to our second research question, we first made a series of correlation analyses between continuous variables. There were few significant correlations between RF and clinical and diagnostic variables, both with respect to MBI-RF and AAI-RF (see **Table 3**). MBI-RF was significantly and positively correlated with the LPFS-BF Self-domain (*r=*0.36, *p*=0.019), indicating more impaired self-reported self-functioning with higher RF. When controlling for age by partial correlation analysis the correlation was 0.39 (*p*=0.013). Further, it was a significant and positive correlation between MBI-RF and age for the first contact with the health care service due to mental health problems (*r*=0.42, *p*=0.006). When controlling for age of the participants at the time of evaluation correlation was 0.32 (*p*=0.047). DERS Clarity had a significant correlation with MBI-RF only when controlling for age (*r*=0.30, *p*=0.057; *r_partial_=*0.37, *p*=0.020), indicating increasingly perceived problems with emotional clarity in participants with higher MBI-RF.

**Table 3:** Pearson correlation coefficients for RF ratings and patient-reported/clinician-rated variables.

	MBI-RF	AAI-RF
Symptom distress		
Depression (PHQ-9)	.113	-.030
Anxiety (GAD-7)	-.122	-.181
Functioning		
Work and social impairment (WSAS)	.232	.034
Level of personality functioning, Total (LPFS-BF Total)	.244	.043
Level of personality functioning, Self (LPFS-BF Self)	.364^*^	.201
Level of personality functioning, Other (LPFS-BF Other)	.074	-.108
Emotional dysregulation (DERS)		
Clarity	.300	.109
Awareness	-.023	.021
Non-acceptance	.197	.202
Goals	.012	-.003
Impulse	.101	.130
Strategies	-.024	-.161
Former treatment experience		
Age first contact health services	.423^**^	.163
Self-harming and suicide attempts		
Self-harm, age first time	.189	.147
Self-harm, number of times	-.095	.070
Self-harm, frequency last six months	-.175	.003
Suicide attempt, age first time	.343	.486^**^
Suicide attempt, number of times	-.097	-.213
Severity of personality difficulties		
Number of PD-criteria	-.047	-.204
Number of BPD-criteria	.084	-.178

^*^p < 0.05, ^**^p < 0.01 (2-tailed).

There were no differences on the RF measures between patients with and without self-harm past six months (Mean AAI-RF 2.32 (SD=0.98) vs 2.44 (SD=1.33), *t*=0.30, *p*=0.764. Mean MBI-RF 1.97 (SD=1.00) vs 2.22 (SD=1.39), *t*=0.61, *p*=0.544), nor between patients with or without suicide attempt past six months (Mean AAI-RF 2.17 (SD=1.84) vs 2.68 (SD=0.80), *t*=1.10, *p*=0.293. Mean MBI-RF 2.33 (SD=1.97) vs 2.25 (SD=0.85), *t*=0.16, *p*=0.873). However, there was significantly higher AAI-RF, but not MBI-RF, among patients with lifetime suicide attempt, compared with those without (Mean AAI-RF 2.57 (SD=1.10) vs 1.92 (SD=0.64), *t*=2.37, *p*=0.023. Mean MBI-RF 2.19 (SD=1.14) vs 1.69 (SD=0.86), *t*=1.52, *p*=0.138).

In order to explore whether there might be different associations with clinical variables among those with totally or almost totally absent RF (i.e., sparsity of mental state information, such as concrete, distorted or evasive explanations) and those with low to ordinary levels of RF, MBI-RF and AAI-RF were dichotomized into “Absent RF” (RF<2) and “Low to ordinary RF” (RF≥2). When dividing the sample into Absent (n=19) or Low to ordinary RF (n=25) by MBI-RF, the results were in line with previous findings, i.e. the Absent-RF group had lower mean scores on the LPFS-BF Self-domain (*t*= 2.31, *p*=0.027), and they were younger at the first contact with the health care service due to mental health problems (*t*=2.19, *p*=0.034). There was also a higher, although non-significant level on DERS Clarity in the Low to ordinary-RF group compared with the Absent-RF group (*t* =1.93, *p*=0.061).

When dichotomizing AAI-RF and dividing the sample into patients with Absent (n=11) or Low to ordinary (n=34) RF, we found significant differences for three variables without corresponding significant correlations for the non-dichotomized AAI-RF. It was a significantly younger age of first suicide attempt in the Absent-RF group (*t*=2.39, *p*=0.024). Further, the Absent-RF group had significantly more suicide attempts than the Low to ordinary-RF group (*t*=2.86, *p*=0.008), and suicide attempts last six months were more frequent in the Absent-RF group than in the Low to ordinary RF-group (*t*=3.90, *p*<0.001).

## Discussion

The primary aim of this study was to investigate the convergent validity of RF ratings based on the MBI by comparing MBI-RF with AAI-RF. The results indicate that the convergent validity is high, suggesting that the MBI-RF and the AAI-RF to a large degree measure the same construct, i.e., reflective functioning, despite a different thematic focus of the two interviews. Few studies have examined convergent validity between different interview-based RF measures, and to the best of our knowledge, there are currently no studies on BPD samples. The finding in the present study is however in line with two studies on non-clinical samples comparing the Brief Reflective Function Interview (BRFI), an abbreviated version of the AAI, with the AAI, with reported coefficients respectively 0.88 (*p<0.001*) and 0.71 (*p<0.01*) (13, 54). Moreover, in a study on patients with obsessive-compulsive disorder symptom-specific RF and AAI-RF were significantly correlated (*r*=0.74, *p*<0.01) (21). Hence, the MBI with its focus on a clinically significant and therapy-relevant aspect of BPD has potential as a tool in assessment of RF in treatment studies on this group of patients.

The finding that mean MBI-RF was slightly lower than AAI-RF is in line with studies comparing symptom-focused RF, like MBI-RF, with general RF like AAI-RF. In a study on panic disorder patients, baseline symptom-specific RF (M=4.43) was significantly lower than general RF (M=5.15, *p*<0.001) (55). Further, in the above-mentioned study on patients with obsessive-compulsive disorder, symptom-specific RF at baseline (M=2.97) was considerably lower than AAI-RF (M=4.32) (21).

Regarding the MBI, an obvious reason for this impairment of RF could be the very fact that the interview investigates a recent episode of mentalizing breakdown, thus potentially leading to a certain reactivation of an emotionally challenging interaction in a close relationship when performing the interview. Both the resulting emotional arousal and the potential hyperactivation of the attachment system can impede mentalizing capacity in the present situation (1). AAI, on the other hand, particularly deals with past and potentially processed experiences, and may consequently to a lesser degree inhibit mentalizing ability during the interview (56). This could imply that MBI-RF is more state-sensitive, in the same manner as session-based RF in psychotherapy, while the AAI is tapping into more stable, trait-like characteristics of RF (57). However, the slightly higher RF values from the AAI could also be due to the possibility of a richer narrative from this extensive interview with its thorough questioning of early attachment relations (54), which could give more evident RFS markers for the RF rating (2).

As to the secondary aim, the main finding is that there were few significant associations between RF ratings (MBI-RF and AAI-RF) and core symptoms of BPD and levels of functional impairment and symptom distress. Hence, with a few exceptions, the present results indicate that measures of MBI-RF and AAI-RF are different from clinical symptoms. This is in line with Müller and colleagues caution that a method for assessment of mentalizing should not be intertwined with the assumed consequences of mentalizing impairment (like emotional dysregulation) (58). And as stated by Kullgard et al. (21) symptom-specific RF and general RF are not targeted to measure the severity of symptoms, but instead to capture the ability to reflect about symptoms and life experiences.

Still, there were a few statistically significant associations between the RF-measures and clinical features. Higher MBI-RF was positively associated with higher age at the first contact with health care services due to mental health problems. It has been hypothesized that RF may serve as a mediator between childhood adversity and the development of personality pathology and psychiatric distress (27), and the finding indicates a possible protective effect of RF. Correspondingly, for AAI-RF there was a significant and positive correlation with age of first suicide attempt. There were also some significant findings for the AAI Absent-RF group, altogether suggesting a potentially protective effect of RF against suicidal behavior. However, this did not apply for MBI-RF, neither as a dichotomized nor continuous variable. And for AAI-RF there was significantly higher RF in the group of patients with lifetime suicide attempts. Thus, based on these contradictory findings doubt can be raised about the protective effect of RF on suicidal behavior, and further studies are needed to clarify the role of interview-based RF in this area.

Some unexpected results were found for MBI-RF. The positive correlations with the LPFS-BF Self-domain and DERS Clarity indicate increasingly perceived impairment with higher RF. We can only speculate, but these counter-intuitive findings could reflect awareness of or increased insight into own symptoms and challenges among those with higher RF (5), while self-reported personality functioning may be more prone to bias among those with lower RF (19). If so, it may raise questions regarding the validity of self-report of various aspects of personality functioning among patients with very low mentalizing capacity. For these patients self-reported behavior (e.g. health service usage and suicidality) could be less susceptible to bias due to its concrete nature (7).

The strengths of the present study include the clinically representative sample of patients with moderate to severe BPD psychopathology, contributing to the ecological validity of the MBI as a BPD-focused RF assessment method. Moreover, in this study a convergent validity measure was applied, i.e. the gold standard AAI, that appropriately supports the validity of the MBI (59). Further, inter-rater reliability is an important quality indicator for observer-based instruments, and in the present study it was satisfactory to good for the MBI-RF ratings (2).

The findings must also be interpreted in light of some important limitations. The small sample size made the results prone to type II errors. However, with some exceptions (3, 5) studies of RF involving AAI are typically based on relatively small samples because of the labor intensive administration, transcribing and coding procedure (9). Another limitation is the relatively narrow range of RF-ratings in the study sample, i.e., from 0 to 5 with the majority of ratings between 1 and 3. Considering that the RFS ranges from -1 to 9, the validity of the MBI for ratings especially in the upper part of the RF Scale thus remains to be established. Nevertheless, the RF-levels of the study sample are representative for patients with moderate to severe BPD psychopathology, which are the target group of the study (3, 26, 60, 61).

Moreover, of the three raters of MBI-RF, two of them (DAU & TW) also carried out all the AAI-RF ratings. Thus, because workgroups may develop idiosyncratic RF rating routines, the convergent validity of MBI-RF would have been strengthened by separate groups scoring respectively MBI-RF and AAI-RF (22). A minority of the AAI-RF ratings were made by TW on interviews she had conducted herself. Even if the timespan between the de-identified transcribed interviews and ratings was long, a potential effect on RF scores cannot be ruled out.

Despite these limitations, the present study provides preliminary support for the convergent validity of the MBI as a BPD-focused RF assessment method as compared with evaluation of RF based on AAI. RF-ratings based on MBI do not overlap with clinical measures of symptoms and functional impairment. The MBI is a half hour and easy to administer interview that has the potential as a time saving method to be applied in treatment research on patients with borderline pathology. Whether MBI-RF is a useful and clinically meaningful tool for studying psychotherapy processes and outcome should be investigated in future studies. Further, even if the MBI is specifically developed for borderline personality pathology, its potential as a RF assessment method in other PDs and for general severity of personality pathology should be subject to future research.

## Data availability statement

The raw data supporting the conclusions of this article will be made available by the authors, without undue reservation.

## Ethics statement

The studies involving human participants were reviewed and approved by the Norwegian Regional Committee for Medical Research. The patients/participants provided their written informed consent to participate in this study.

## Author contributions

DU: Conceptualization, Data curation, Formal Analysis, Investigation, Methodology, Visualization, Writing – original draft, Writing – review & editing. MJ: Data curation, Writing – review & editing. EK: Conceptualization, Writing – review & editing. GP: Conceptualization, Formal Analysis, Methodology, Visualization, Writing – review & editing. TW: Conceptualization, Data curation, Investigation, Methodology, Project administration, Supervision, Writing – review & editing.
