# Corrigendum: Minding mentalizing – convergent validity of the Mentalization Breakdown Interview

**DOI:** 10.3389/fpsyt.2024.1479612

**Published:** 2024-08-21

**Authors:** Dag Anders Ulvestad, Merete Selsbakk Johansen, Elfrida Hartveit Kvarstein, Geir Pedersen, Theresa Wilberg

**Affiliations:** ^1^ Outpatient Clinic for Specialized Treatment of Personality Disorders, Section for Personality Psychiatry and Specialized Treatments, Department for National and Regional Functions, Division of Mental Health and Addiction, Oslo University Hospital, Oslo, Norway; ^2^ Institute of Clinical Medicine, University of Oslo, Oslo, Norway; ^3^ South-Eastern Norway Regional Health Authority, Oslo, Norway; ^4^ Section for Personality Psychiatry and Specialized Treatments, Department for National and Regional Functions, Division of Mental Health and Addiction, Oslo University Hospital, Oslo, Norway; ^5^ Network for Personality Disorders, Section for Personality Psychiatry and Specialized Treatments, Department for National and Regional Functions, Division of Mental Health and Addiction, Oslo University Hospital, Oslo, Norway; ^6^ Institute of Basic Medical Sciences, University of Oslo, Oslo, Norway; ^7^ Section for Treatment Research, Department for Research and Innovation, Division of Mental Health and Addiction, Oslo University Hospital, Oslo, Norway

**Keywords:** borderline personality disorder, mentalizing, mentalization breakdown, reflective functioning, assessment method, validation

In the published article, there was an error in the Affiliations for Dag Anders Ulvestad. As well as having affiliation 1, he should also have affiliation 2 ‘Institute of Clinical Medicine, University of Oslo, Oslo, Norway’.

The authors apologize for this error and state that this does not change the scientific conclusions of the article in any way. The original article has been updated.

